# Metabolic and Transcriptomic Signatures of the Acute Psychological Stress Response in the Mouse Brain

**DOI:** 10.3390/metabo13030453

**Published:** 2023-03-20

**Authors:** Haein Lee, Jina Park, Seyun Kim

**Affiliations:** 1 Department of Biological Sciences, Korea Advanced Institute of Science and Technology (KAIST), Daejeon 34141, Republic of Korea; 2 KAIST Institute for the BioCentury, Korea Advanced Institute of Science and Technology (KAIST), Daejeon 34141, Republic of Korea; 3 KAIST Stem Cell Center, Korea Advanced Institute of Science and Technology (KAIST), Daejeon 34141, Republic of Korea

**Keywords:** stress, brain, transcriptomics, metabolomics, metabolism

## Abstract

Acute stress response triggers various physiological responses such as energy mobilization to meet metabolic demands. However, the underlying molecular changes in the brain remain largely obscure. Here, we used a brief water avoidance stress (WAS) to elicit an acute stress response in mice. By employing RNA-sequencing and metabolomics profiling, we investigated the acute stress-induced molecular changes in the mouse whole brain. The aberrant expression of 60 genes was detected in the brain tissues of WAS-exposed mice. Functional analyses showed that the aberrantly expressed genes were enriched in various processes such as superoxide metabolism. In our global metabolomic profiling, a total of 43 brain metabolites were significantly altered by acute WAS. Metabolic pathways upregulated from WAS-exposed brain tissues relative to control samples included lipolysis, eicosanoid biosynthesis, and endocannabinoid synthesis. Acute WAS also elevated the levels of branched-chain amino acids, 5-aminovalerates, 4-hydroxy-nonenal-glutathione as well as mannose, suggesting complex metabolic changes in the brain. The observed molecular events in the present study provide a valuable resource that can help us better understand how acute psychological stress impacts neural functions.

## 1. Introduction

Stress is defined as the related reactions of the body that are triggered by various environmental factors, which can be traced back to the general adaptation syndrome proposed by Selye [1,2,3]. The acute stress (AS) response promotes the chances of survival by regulating an organism’s energy resources to meet the metabolic demands [4]. When the stressor has lessened, stress must be immediately managed to avoid stress-related pathological responses [5]. Remarkably, organisms can efficiently react to stressful conditions and maintain mental health, even in response to severe stressors [6,7,8]. However, stress overload potentially disrupts physiological homeostasis, damages certain brain areas, and can develop into many mental diseases such as depression, anxiety, and post-traumatic stress disorder (PTSD) [9,10,11].

Anatomical, physiological, and behavioral studies conducted over the past few decades have provided key insights into the mechanisms underlying stress-induced changes in the brain. For example, mood disorders have been linked to the hypothalamic–pituitary–adrenal cortex (HPA) axis dysregulation caused by stress [12]. Moreover, many studies have demonstrated that increases in glucocorticoid levels can affect the morphology and function of the brain [13,14,15]. Stress-induced changes in neurotransmitters, neuropeptides, and neurotrophic factors have also been widely reported in the brain [15,16,17]. Additionally, increases in the activity and synaptic plasticity of the amygdala are considered as another important driving factor of stress-induced mood disorders [18,19,20,21,22,23].

Several studies have explored stress-mediated molecular alterations in the brain [24,25,26,27,28,29,30,31,32,33]. Some studies have demonstrated transcriptional changes at different time points after stress challenge [25,34,35,36,37,38]. Other omics-based approaches (e.g., proteomics, metabolomics) have often been adopted to explore stress-linked molecular alterations in the brain. Various stress paradigms such as chronic social defeat stress, prenatal stress, and restraint have been used to identify molecular changes in different brain regions, classically associated with anxiety and depression-related behaviors including the prefrontal cortex, hippocampus, amygdala, and nucleus accumbens [33,39,40,41,42,43,44,45,46,47,48]. However, our knowledge on the biochemical and molecular changes occurring in the brain in response to acute stress remains largely insufficient.

Here, we employed a multi-omics approach in mice to identify core differentially expressed molecules and pathways underlying acute psychological stress. The acute water avoidance assay was used to induce anxiety-related phenotypes and to identify molecular markers and related pathways. Afterward, RNA-sequencing and metabolomics analyses were respectively conducted to detect differences in the gene expression and metabolite profiles of the whole brain of stress-exposed and control mice. Intriguingly, we detected alterations in metabolites and transcriptional reprogramming, providing insights into the genetic and metabolic changes linked to the psychological responses to acute stress.

## 2. Materials and Methods

### 2.1. Animals

C57BL/6J male mice were used for the experiments (8–10-week-old; KRIBB, Cheongju, Republic of Korea). Mice were housed under specific pathogen-free conditions in a 12 h light–dark schedule, and their food and water were provided ad libitum. Animal protocols were performed in accordance with guidelines approved by the Korea Advanced Institute of Science and Technology Animal Care and Use Committee.

### 2.2. Water Avoidance Stress Procedure

The 1 h water avoidance stress (WAS) procedure consisted of placing each mouse on a small platform (3 cm width × 5 cm length × 4 cm height) in the middle of a small plastic basin (40 cm length × 40 cm width × 60 cm height) filled with warm water (22 °C) at the height level of the platform. Control group mice were placed on the same platform but in a waterless container for 1 h. All animals were sacrificed immediately after completion of the stress procedure. After removing the olfactory bulbs and cerebellum, the whole forebrain was separated into two hemispheres. Each hemisphere was subject to RNA-Seq and metabolomics analysis.

### 2.3. Total RNA Extraction and RNA-Sequencing

Total RNA was isolated using Trizol reagent (Invitrogen, Waltham, MA, USA). RNA quality was assessed with an Agilent 2100 bioanalyzer using the RNA 6000 Nano Chip (Agilent Technologies, Santa Clara, CA, USA). RNA quantification was performed using an ND-2000 Spectrophotometer (Thermo Fisher Scientific, Inc., Waltham, MA, USA). QuantSeq 3′ mRNA-Seq was performed by Ebiogen (Ebiogen, Inc., Seoul, South Korea). The library of control and test RNAs were constructed by using the QuantSeq 3′ mRNA-Seq Library Prep Kit (Lexogen GmbH, Vienna, Austria) according to the manufacturer’s instructions. In brief, each prepared 500 ng total RNA were hybridized with an oligo-dT primer containing an Illumina-compatible sequence at its 5′ end. Then, reverse transcription was performed. After degradation of the RNA template, second strand synthesis was initiated by random primers with an Illumina-compatible linker sequence at the 5′ end. The double-stranded library was purified by removing all reaction components using magnetic beads. After the library was amplified to add the complete adapter sequences required for cluster generation, it was purified from the PCR components. High-throughput sequencing was performed as single-end 75 bp sequencing using NextSeq 500 (Illumina, Inc., San Diego, CA, USA).

QuantSeq 3′ mRNA-Seq reads were aligned using Bowtie2 [49]. Bowtie2 indices were generated from genome assembly sequences or representative transcript sequences for alignment to the genome and transcriptome. The alignment file was used to assemble transcripts, estimate their abundances, and detect differential gene expression. Based on counts from unique and multiple alignments using coverage in Bedtools [50], differentially expressed genes were determined. The RC (read count) data were processed based on the quantile normalization method using EdgeR within R (R Development Core Team, 2016) using Bioconductor [51]. Gene classification was based on searches conducted by the DAVID (http://david.ncifcrf.gov/ (accessed on 17 March 2023)) and Medline databases (http://www.ncbi.nlm.nih.gov/ (accessed on 17 March 2023)). Data mining and graphic visualization were performed using ExDEGA (Ebiogen Inc., Seoul, South Korea). A cutoff was applied at a normalized gene expression (log2) of 2. Sequencing data were deposited in the NCBI GEO (accession number GSE227416).

### 2.4. Global Metabolite Profiling and Data Analysis

The sample preparation was carried out as described previously at Metabolon, Inc. (Morrisville, NC, USA) [52,53]. Briefly, samples were prepared using the automated MicroLab STAR^®^ system (Hamilton Company, Reno, NV, USA). A recovery standard was added prior to the first step in the extraction process for QC purposes. To remove protein, dissociate small molecules bound to protein or trapped in the precipitated protein matrix, and to recover chemically diverse metabolites, proteins were precipitated with methanol under vigorous shaking for 2 min (Glen Mills GenoGrinder 2000, Glen Mills, Inc., Clifton, NJ, USA) followed by centrifugation. The resulting extract was divided into five fractions: one for analysis by UPLC-MS/MS with positive ion mode electrospray ionization, one for analysis by UPLC-MS/MS with negative ion mode electrospray ionization, one for analysis by UPLC-MS/MS polar platform (negative ionization), one for analysis by GC-MS, and one sample was reserved for backup. Samples were placed briefly on a TurboVap^®^ (Zymark, Hopkinton, MA, USA) to remove the organic solvent. For LC, the samples were stored overnight under nitrogen before preparation for analysis. For GC, each sample was dried under vacuum overnight before preparation for analysis.

Several types of controls were analyzed in concert with the experimental samples: a pooled matrix sample generated by taking a small volume of each experimental sample (or alternatively, use of a pool of well-characterized human plasma) served as a technical replicate throughout the dataset; extracted water samples served as process blanks; and a cocktail of QC standards that were carefully chosen not to interfere with the measurement of endogenous compounds were spiked into every analyzed sample, allowed for instrument performance monitoring, and aided in chromatographic alignment.

Metabolomic analyses were conducted by Metabolon, Inc. using ultra-high-performance liquid chromatography/tandem mass spectrometry (UHPLC/MS/MS), as previously described [54]. The LC/MS portion of the platform was based on a Waters ACQUITY UPLC and a Thermo Scientific Q-Exactive high resolution/accurate mass spectrometer interfaced with a heated electrospray ionization (HESI-II) source and Orbitrap mass analyzer operated at a 35,000 mass resolution. The sample extract was dried then reconstituted in acidic or basic LC-compatible solvents, each of which contained eight or more injection standards at fixed concentrations to ensure injection and chromatographic consistency. One aliquot was analyzed using acidic positive ion optimized conditions and the other using basic negative ion optimized conditions in two independent injections using separate dedicated columns (Waters UPLC BEH C18-2.1 × 100 mm, 1.7 μm). Extracts reconstituted in acidic conditions were gradient eluted from a C18 column using water and methanol containing 0.1% formic acid. The basic extracts were similarly eluted from C18 using methanol and water, however, with 6.5 mM ammonium bicarbonate. The third aliquot was analyzed via negative ionization following elution from a HILIC column (Waters UPLC BEH Amide 2.1 × 150 mm, 1.7 μm) using a gradient consisting of water and acetonitrile with 10 mM ammonium formate. The MS analysis alternated between MS and data-dependent MS2 scans using dynamic exclusion, and the scan range was from 80 to 1000 *m*/*z*. The samples destined for analysis by GC-MS were dried under vacuum for a minimum of 18 h prior to being derivatized under dried nitrogen using bistrimethyl-silyltrifluoroacetamide. Derivatized samples were separated on a 5% diphenyl/95% dimethyl polysiloxane fused silica column (20 m × 0.18 mm ID; 0.18 μm film thickness) with helium as the carrier gas and a temperature ramp from 60 °C to 340 °C in a 17.5 min period. Samples were analyzed on a Thermo-Finnigan Trace DSQ fast-scanning single-quadrupole mass spectrometer using electron impact ionization (EI) and operated at unit mass resolving power. The scan range was from 50 to 750 *m*/*z*.

The informatics system was composed of the Laboratory Information Management System (LIMS), the data extraction and peak-identification software, data processing tools for QC and compound identification, and a collection of information interpretation and visualization tools for use by data analysts. Using the hardware and software of Metabolon, the extraction of raw data, the identification of peaks, and QC processing were performed. These systems were built on a web-service platform that utilizes Microsoft’s .NET technologies. Compounds were identified by comparing them to library entries of purified standards or recurrent unknown entities. Metabolon’s library is maintained based on authenticated standards that include retention time/index (RI), the mass-to-charge ratio (*m*/*z*), and chromatographic data (including MS/MS spectral data) for all molecules present in the library. Additionally, biochemical identifications were based on the retention index within a narrow RI window of the proposed identification, accurate mass match to the library +/− 10 ppm, and the MS/MS forward and reverse scores between the experimental data and authentic standards. MS/MS scoring was based on a comparison of ions present in the experimental and library spectrum. Based on one of these factors, there may be similarities between these molecules, however, the use of all three data points can be utilized to distinguish and differentiate biochemicals. More than 3300 commercially available purified standard compounds were collected and registered in LIMS for analysis on all platforms for the determination of their analytical characteristics. Additional mass spectral entries were created for structurally unnamed biochemicals, which were identified by virtue of their recurrent nature in both chromatographic and mass spectra.

Metabolon ensured the accurate and consistent identification of true chemical entities by removing those representing the system artifacts, misassignments, and background noise through QC and curation processes. For each compound, library matches were confirmed for each sample and corrected if necessary. Metabolite peaks were quantified using the area under the curve. If the measurements were conducted for more than one day, a normalization step was performed. Each compound was modified in the run-day blocks by normalizing each data point proportionally by registering the median at one (1.00). Statistical calculations were carried out by performing Welch’s two-sample *t*-test.

## 3. Results

### 3.1. Transcriptome Analysis of the Brain under Acute Stress

**Figure 1:**
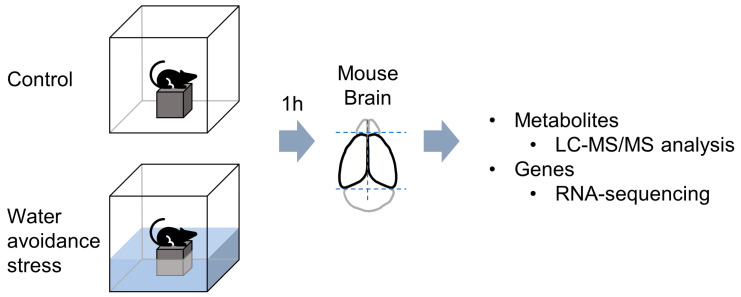
Scheme of acute water avoidance stress (WAS) exposure; brain tissue samples were examined at 1 h after WAS by untargeted metabolomics (*n* = 6) and bulk RNA-sequencing analysis (*n* = 3).

**Figure 2:**
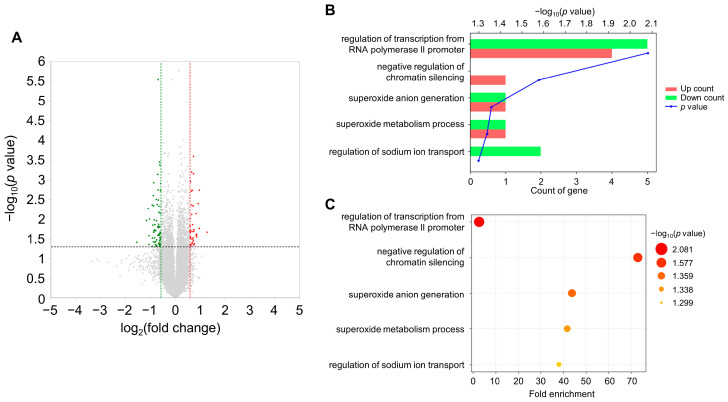
Effects of acute WAS on the transcriptome of the mouse whole brain. (**A**) Volcano plot of the differentially expressed genes between the water-stressed group and control group. The green dots indicate genes that reduced 1.5-fold below, and the red dots indicate genes that increased 1.5-fold above. The vertical dotted lines showed 1.5-fold changes. The dotted black-line shows the *p* value cutoff (*p* = 0.050) with points above the line having *p* < 0.050 and points below the line having *p* > 0.050. (**B**,**C**) Gene Ontology (GO) enrichment analysis of the differentially expressed genes (DEGs) between the water-stressed and control groups. GO categories of the biological process are shown to the left of the charts. (**B**) Count of upregulated (red bars) and downregulated (green bars) genes were represented. The blue line of the graphs means −log_10_(*p* value). (**C**) The *x*-axis represents the fold enrichment of the GO terms. The color and the size of the bubbles depend on the *p* value.

The water avoidance stress model was adopted to induce acute psychological stress, which strongly activates the sympathetic nervous system and the hypothalamic–pituitary–adrenal (HPA) axis. Adult male mice were subjected to water stress for 1 h at room temperature (Figure 1). To gain a deeper understanding of the molecular mechanisms occurring in the stress-exposed brain, forebrain tissues were used for RNA-Seq analyses. A total of only 60 (25 upregulated and 35 downregulated) differentially expressed genes (DEGs) were identified between the control and AS-exposed animals based on a fold change (FC) ≥1.5 for upregulation and ≤−1.5 for downregulation (Figure 2A and Supplementary Table S1). Once we had obtained a list of DEGs, GO enrichment analysis was conducted to clarify the biological functions in the mouse brain affected by acute water avoidance stress. We identified several modestly but significantly enriched GO categories including “regulation of transcription from RNA polymerase II promoter”, “negative regulation of chromatin silencing”, “superoxide anion generation”, “superoxide anion metabolism”, and “regulation of sodium ion transport” in the stress-exposed brain (Figure 2B,C). Downregulation of Nos1 (nitric oxide synthase 1, neuronal NOS) was notable since neuronal NOS catalyzes the synthesis of nitric oxide (NO), a potent neuromodulator, implying perturbed neuronal communication in response to acute WAS. Changes in regulatory Ncf1/p47phox and Noxo1 subunits in superoxide-generating NADPH oxidase also suggest the dysregulation of ROS metabolism under acute WAS.

### 3.2. Unique Alterations in a Broad Range of Metabolites in the Mouse Brain Exposed to Acute Water Avoidance Stress

**Figure 3:**
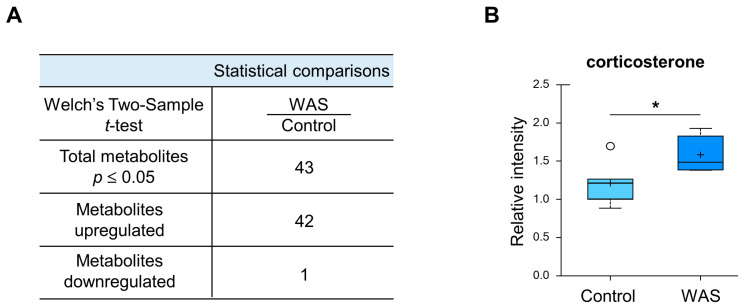
Overview on altered metabolites and directional changes. (**A**) Number of significantly altered biochemicals in the brain samples. (**B**) Box plot for corticosterone. The boxes outline the second and third quartile (middle 50%) of the data for each sample type. The error bars on the graph represent 1.5 × IQR (interquartile range for that metabolite within the sample type. * *p* < 0.05 (Welch’s two-tailed *t*-test).

To identify stress-induced metabolic changes, we next performed global untargeted metabolomics analysis and found that 43 metabolites out of the 407 identified in total were significantly different after 1 h of stress conditions (Figure 3A and Supplementary Table S2). Notably, the stress group displayed elevated levels of corticosterone, a representative stress biomarker belonging to the glucocorticoid class of steroid hormones [55], which explained the activation of the psychological stress response upon exposure to the water avoidance stress challenge (Figure 3B). Different metabolites in stressed brain samples fell into the following broad categories: lipid metabolism, endocannabinoid metabolism, amino acid metabolism, and additional miscellaneous categories.

### 3.3. Activation of Lipolysis and Eicosanoid Metabolic Pathways

**Figure 4:**
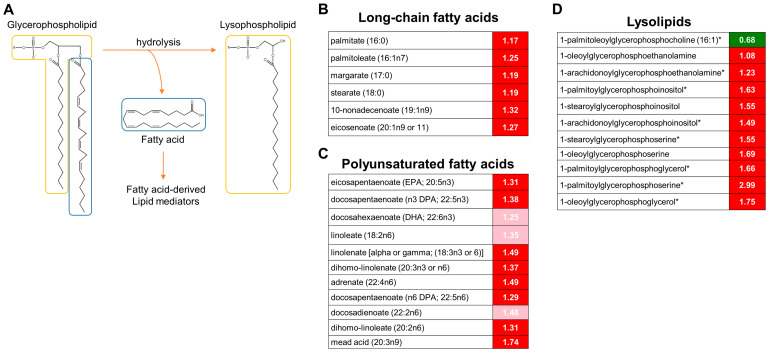
Accumulation of long-chain fatty acids and polyunsaturated fatty acids by acute WAS. (**A**) Scheme of the PLA2-dependent lipolysis. Measurements of brain content of long-chain fatty acids (**B**), PUFAs (**C**), and lysolipids (**D**). Significantly different values determined by Welch’s two-tailed *t*-test (*p* < 0.05). Green color indicates significant difference (*p* ≤ 0.05) between the groups shown, metabolite ratio of < 1.00, Red indicates significant difference (*p* ≤ 0.05) between the groups shown; metabolite ratio of ≥ 1.00, Light Red indicates narrowly missed statistical cutoff for significance 0.05 < *p* < 0.10, metabolite ratio of ≥ 1.00. * Indicates compounds that have not been officially confirmed based on a standard, but identified by virtue of their recurrent chromatographic and spectral nature.

**Figure 5:**
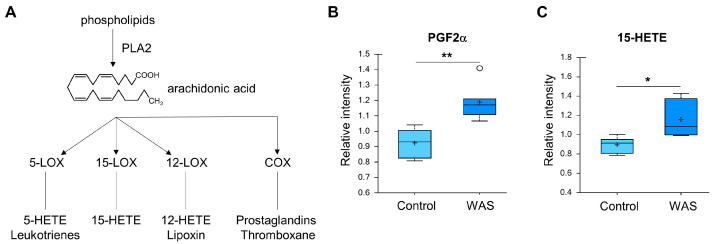
Effects of acute WAS on eicosanoids. (**A**) Eicosanoid metabolism. Measurements of brain content of PGF2alpha (**B**) and 15-HETE (**C**). * *p* < 0.05, ** *p* < 0.01 (Welch’s two-tailed *t*-test).

When the stress group was compared to the control group, significant differences were mostly observed in lipid metabolism. Most notably, the stress group exhibited elevated levels of long-chain fatty acids and polyunsaturated fatty acids (PUFAs). In fact, 14 out of the 43 statistically significant differences between the stress and control groups involved these metabolites (Figure 4B,C). In addition to free fatty acids, lysolipids were markedly increased in the stressed brain tissues (Figure 4D). Lysolipids are formed when lipases in the cell remove fatty acid moieties from the Sn-1 or Sn-2 position of phospholipids [56]. Most of the lysolipids that were elevated in this study lacked acyl chains at the Sn-2 position and were derived from phosphatidylinositol (PI), phosphatidylserine (PS), and phosphatidylglycerol (PG). Taken together, these changes in lipid metabolites suggest that there was an increase in phospholipase A2 (PLA2) activity. PLA2 specifically cleaves fatty acids at the Sn-2 position. Unsaturated fats such as arachidonic acid are commonly found at the Sn-2 position of phospholipids. Therefore, increases in phospholipase A2 activity can result in arachidonate release and alterations in the eicosanoid signaling pathway [57]. The levels of two eicosanoid metabolites including prostaglandin F2alpha (PGF2alpha) and 15-hydroxyeicosatetraenoic acid (15-HETE) (Figure 5) were elevated in the brain of the stressed mice.

### 3.4. Acute Stress Elevates Endocannabinoid Levels in the Brain

**Figure 6:**
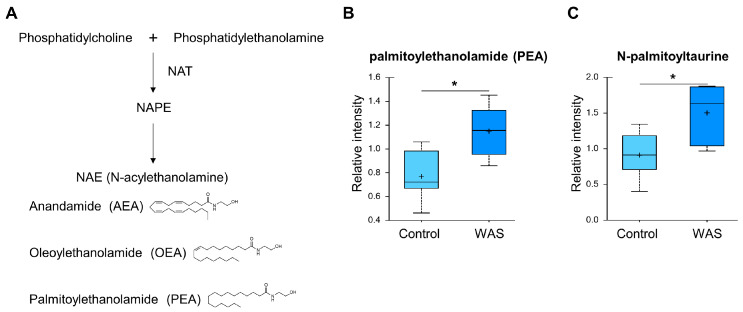
Effects of acute WAS on the endocannabinoid levels. (**A**) Endocannabinoid metabolism. Measurements of brain content of palmitoylethanolamide (**B**) and N-palmitoyl taurine long-chain fatty acids (**C**). * *p* < 0.05 (Welch’s two-tailed *t*-test).

Elevated levels of palmitoylethanolamide (PEA) and N-palmitoyl taurine were detected in the brains of the stressed animals (Figure 6). These metabolites are classified as endocannabinoids, which are lipid signaling molecules that regulate a wide range of physiological responses including mood, appetite, nociception, and memory [58]. PEA is reported to have anti-inflammatory and neuroprotective properties [59]. N-acyl taurines such as N-palmitoyl taurine are known to activate TRP receptors, which play diverse roles in processes such as mechanical transduction, osmotic regulation, and temperature sensing [60].

### 3.5. Changes in Amino Acid Metabolism

**Figure 7:**
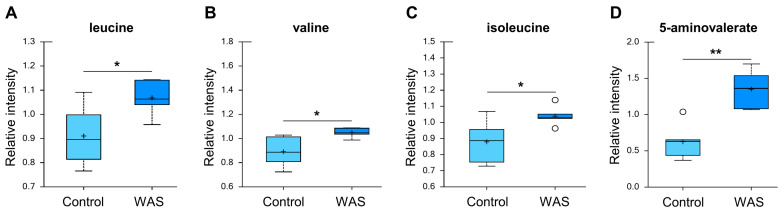
Effects of acute WAS on branched amino acids and lysine metabolism. Measurements of the brain content of branched amino acids (**A**–**C**) and 5-aminovalerate (**D**). * *p* < 0.05, ** *p* < 0.01 (Welch’s two-tailed *t*-test).

The stress-exposed group displayed elevated levels of all three branched-chain amino acids (BCAAs) including leucine, isoleucine, and valine (Figure 7). The changes in these amino acids reflect either increased protein breakdown within brain cells (e.g., neurons, glia) or increased uptake of amino acids into the brain from the bloodstream. Among other amino acid metabolites, significant increases in the lysine catabolite 5-aminovalerate were also found in stressed brain tissues (Figure 7), indicating alterations in the utilization, catabolism, or uptake of lysine.

### 3.6. Increased 4-Hydroxy-Nonenal-Glutathione Levels

**Figure 8:**
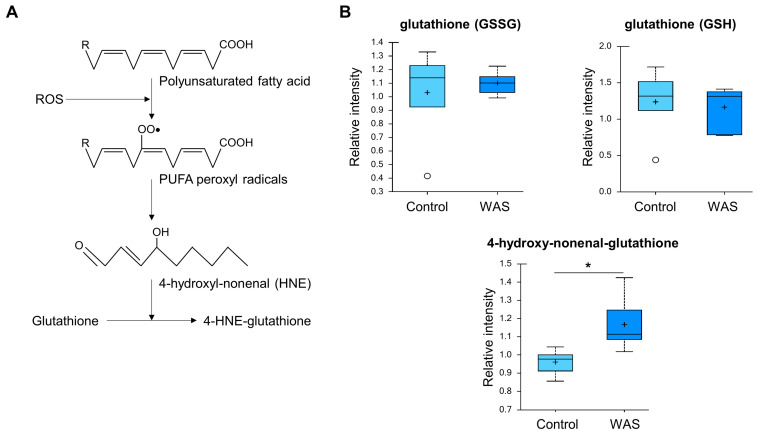
Increased 4-hydroxy-nonenal-glutathione levels by acute WAS. (**A**) Scheme of 4-hydroxy-nonenal-glutathione synthesis. Measurements of brain content of 4-hydroxy-nonenal-glutathione (**B**). * *p* < 0.05 (Welch’s two-tailed *t*-test).

Higher levels of 4-hydroxy-nonenal-glutathione were detected in the stress group (Figure 8). This metabolite is a glutathione conjugate of 4-hydroxy-2-nonenal (HNE), a major metabolite produced from the oxidation of n-6 PUFAs such as linoleic, γ-linolenic, or arachidonic acids [61]. HNE is known to react with a large number of macromolecules such as proteins, thus contributing to protein cross-linking and inducing carbonyl stress [61]. Given that 4-hydroxy-nonenal-glutathione is produced from the detoxification process, elevated 4-hydroxy-nonenal-glutathione can be interpreted as a metabolic signature of oxidative stress triggered by the acute water avoidance conditions.

### 3.7. Increased Mannose Levels

**Figure 9:**
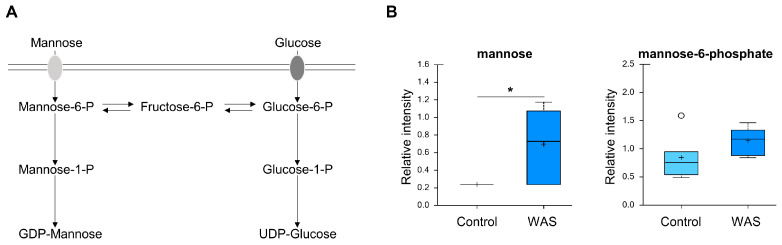
Effects of acute WAS on mannose levels. (**A**) Mannose metabolism. Measurements of brain content of mannose (**B**). * *p* < 0.05 (Welch’s two-tailed *t*-test).

We found no apparent changes in the major metabolites from carbohydrate metabolism (e.g., glycolysis, TCA cycle, pentose phosphate pathway). The levels of other carbohydrates such as fructose, sorbitol, galactose 1-phosphate, and galactonate were similar between the control and acute stress samples. Interestingly, in contrast to the control brain, the brain tissues of the stressed mice exhibited a nearly 3-fold significant increase of mannose, but not its immediate downstream product, mannose 6-phosphate (Figure 9). This selective increase suggested that robust mannose uptake occurs in the brain during the early acute phase of psychological stress.

### 3.8. Integration Analysis of Transcriptomic and Metabolomic Data

**Figure 10:**
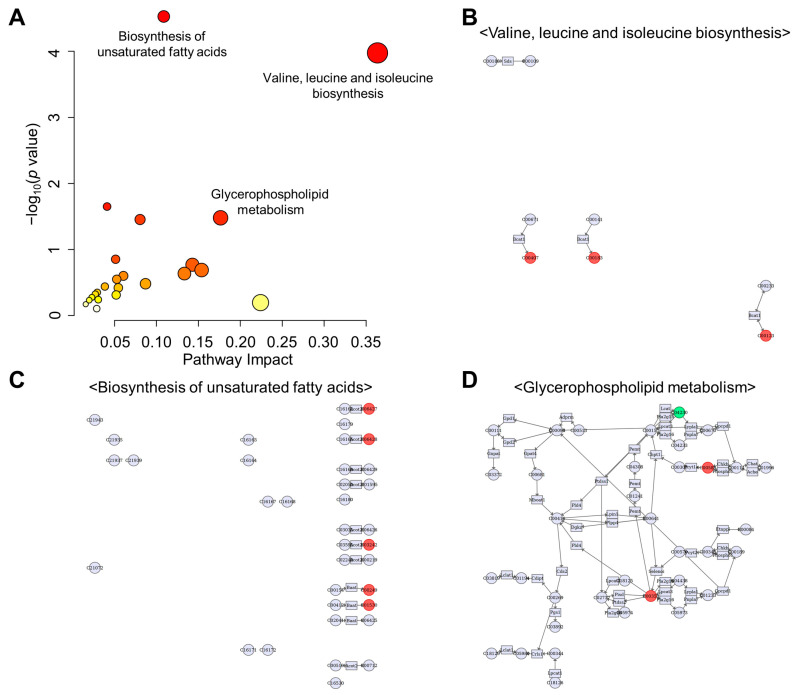
Results of the joint-pathway analysis. (**A**) The pathways were shown as a scatter plot from integrating transcriptomics and targeted metabolomics data using MetaboAnalyst 5.0. Pathway impact scores, which summarize normalized topology measures of perturbed genes/metabolites in each pathway is shown on the *x*-axis. −log_10_(*p* value) of the enrichment analysis results is shown on the *y*-axis. The sizes of the data points are correlated with pathway impact scores, and the color gradients correspond to the enrichment analysis results. Pathways were annotated by numbering when the *p* values calculated from the enrichment analysis were ≤0.05. (**B**–**D**) Annotated KEGG pathways. Genes: rectangles, compounds: circles, matching nodes are negative: green, positive: red, based on log(fold change).

Multiple-omics analysis profiling is a comprehensive approach that can significantly increase the strength of results, providing in-depth insights into the molecular mechanisms of biological phenomena compared to transcriptomic, proteomic, or metabolomic analyses alone [62]. Therefore, we used MetaboAnalyst 5.0 to perform the joint pathway analysis of transcriptomic and metabolomic data. The pathway analysis results with MetaboAnalyst 5.0 are shown in Supplementary Table S3. The mRNA enrichment and metabolic pathway analyses revealed significantly altered pathways at both the metabolomic and mRNA expression levels in the acute WAS-exposed mouse brain. Particularly, the dysregulated pathways were involved in the biosynthesis of unsaturated fatty acids, aminoacyl-tRNA biosynthesis, and glycerophospholipid metabolism, among other processes (Figure 10). Valine/leucine/isoleucine biosynthesis was the most impactful pathway showing the highest impact coefficients > 0.35, indicating that BCAA metabolism was dysregulated due to acute WAS treatment.

## 4. Discussion

The stress response is a vital biological function that is crucial for increasing the host’s chances of survival by coordinating energy homeostasis and various other physiological responses. Therefore, the host organism must control its stress levels to avoid the physiological disturbances that accompany chronic stress exposure. Furthermore, excessively intense or frequent stress can impair the homeostatic stress response, leading to the development of psychiatric conditions such as depression, anxiety, and PTSD [9,10,11]. Given the profound role of the brain as the central organ for stress sensing and response as well as the regulation of systemic stress, the goal of this study was to define the major molecular changes of stress-associated genes and metabolites in an unbiased manner. Previous rodent studies have been predominantly conducted under repeated or chronic stress conditions [27,28,32,36,39,42,43,47]. However, compared to our understanding of the effects of chronic stress, the impacts of acute stress (AS) on the brain remain largely unexplored. In addition to the duration of stress, various stressors engage different neuronal populations and circuits. Forced swimming, constraint, or social defeat models are commonly employed in stress response analysis. Here, we chose to focus on the water avoidance stress (WAS) response, a well-known animal assay that mimics the psychological changes in humans in response to environmental stressors.

In this study, 60 mRNAs changed significantly in the mouse brain in response to WAS exposure including 25 mRNAs that increased and 35 that decreased. In our analysis of differentially expressed mRNAs, regulation of transcription from RNA polymerase II promoter, negative regulation of chromatin silencing, superoxide anion metabolism, and the regulation of sodium ion transport were enriched. No genes related to energy metabolism were altered. Instead, levels of metabolic genes such as glutathione S-transferase mu6, Ncf1/p47phox, and Noxo1 were affected by acute WAS, indicating oxidative stress response upon acute WAS. Although there was no apparent change in well-known immediate early genes (e.g., c-jun, c-fos, psd95, homer), our data revealed that acute water avoidance stress regulates the expression of unique sets of genes (e.g., P2Y purinergic receptor, potassium voltage-gated channel subfamily H, member 7 (Kcnh7), neuronal NOS, IGF-BP) in pathways related to synaptic signaling and neuronal activities. Taken together with the altered mRNA levels of Doc2b and calcium binding protein 1 (CaBP1) responsible for calcium-sensitive neuronal activities, these findings clearly suggest potent changes in neuronal communication in stressed brain tissues. Acute WAS also made a robust impact on genes responsible for brain development and morphogenesis (e.g., Satb2, Hist1h1e, Npas1), suggesting the contribution of acute WAS to the accompanied neural defects.

In our analyses, the most striking molecular changes observed in the stress-exposed brain tissues were marked increases in the levels of n-3 and n-6 PUFAs (e.g., linolenate, eicosapentaenoate, and docosapentaenoate) as well as long-chain fatty acids such as palmitoleate and eicosenoate. No changes in the short and medium chain fatty acids were found between the control and stress groups. Furthermore, we found that lysophospholipids lacked acyl chains at the Sn-2 position, which constituted another major change in fatty acid metabolism. These findings strongly suggest that acute WAS conditions trigger the selective activation of PLA2 in the brain. PLA2 is a diverse group of enzymes including secretory PLA2 (sPLA2), cytosolic PLA2 (cPLA2), and calcium-independent PLA2 (iPLA2). In addition to neurotrophic and excitatory signals (e.g., glutamate), inflammatory cytokines are known to activate PLA2 and produce free fatty acids and lysophospholipids [63]. Particularly, the released arachidonic acid is metabolized into bioactive eicosanoids (prostaglandins, leukotrienes, thromboxanes), and lysophospholipids are converted into platelet-activating factors. As expected, the levels of PGF2alpha and 15-HETE were increased in the AS-exposed brain samples. These lipid mediators play critical roles in the initiation, maintenance, and modulation of inflammation and oxidative stress in the brain [64]. It should also be noted that lysophospholipids also act as potent bioactive signaling molecules by binding to their cognate receptors to mediate various cellular activation (e.g., proliferation, migration) and inflammation [56,65]. Another important outcome of PLA2 activation is the accumulation of reactive fatty acid metabolites, which lead to the generation of 4-hydroxy-2-nonenol (HNE). Elevated levels of 4-hydroxy-nonenal-glutathione were also found in the brains of the AS-exposed mice. Collectively, these metabolic signature profiles indicate that acute water avoidance stress stimulates PLA2 and the subsequent release of bioactive lipids (e.g., eicosanoids), which in turn triggers a wide range of signaling events (e.g., neuroinflammation), membrane structural damages, free radical generation, and cellular injury in the brain. Furthermore, there were no differences in the expression levels of PLA2 and other genes (e.g., lipoxygenase, cyclooxygenase) between the stressed and control groups, indicating that these metabolite changes are independent of the transcriptional regulation of the main catalytic enzymes. Similar to our findings, a previous study also reported PLA2 hyperactivation in the rat brain after four weeks of chronic unpredictable stress [66].

Endocannabinoid (eCB) signaling has been interpreted as a gatekeeper against stress and anxiety due to its modulatory role in the behavioral responses to environmental cues [58,67]. Under resting conditions, the eCB anandamide (N-arachidonoylethanolamine; AEA) appears to inhibit the activation of the brain (e.g., amygdala) and the hypothalamic–pituitary–adrenal axis. Glucocorticoid hormones secreted during stress start triggering an array of physiological adaptive events. Endocannabinoid signaling further sends feedback signals to modulate the activity of the hypothalamic–pituitary–adrenal (HPA) axis, which governs the secretion of glucocorticoids [68]. Exposure to acute stress has been known to rapidly reduce or elevate eCB in different brain regions in response to an array of psychological stressors. For example, in response to stressful stimuli, the amygdala shows dynamic changes in eCB levels, resulting in rapid AEA declines by increasing the activity of fatty acid amide hydrolase (FAAH), an AEA-hydrolyzing enzyme [69]. Many other studies have suggested that stress increases 2-arachidonoyl glycerol (2-AG) signaling [70,71]. Elevated levels of the two eCBs, palmitoyl ethanolamide (PEA) and N-palmitoyl taurine, were detected in the AS-exposed brain in our study, thus confirming the well-known cortisol-mediated neuronal activity induced by 1 h of acute WAS stimulation. These data suggest that hyperactive PLA2 metabolism and increased PUFAs (e.g., palmitate) are metabolically connected and contribute to the synthesis and activity of cortisol during the acute stress response.

Unexpectedly, energy and glucose metabolism such as glycolysis, TCA cycle, and oxidative phosphorylation remained unchanged in the brain after 1 h of acute WAS stimulation. The only unique and substantial result from carbohydrate metabolites is the nearly 3-fold increase in mannose levels but no other carbohydrates (e.g., fructose, sorbitol, galactose 1-phosphate, galactonate). Mannose, a C-2 epimer of glucose, is the major monosaccharide component of N-glycans and is internalized into mammalian cells via membrane glucose transporters. Upon its uptake into the cells, D-mannose is phosphorylated by hexokinase and produces mannose-6-phosphate (M6P), which can be directed into the glycolysis or glycosylation pathway. M6P metabolite levels were not altered by AS and our RNA-Seq analyses did not detect the expression of glucose transporter genes, suggesting that the marked increase in mannose levels observed in our study was likely caused by an increased uptake from the peripheral tissues. A previous study demonstrated that plasma concentrations of mannose were positively correlated with insulin resistance, and this effect was independent of the body mass index (BMI) [72]. Indeed, an elevated plasma mannose level is considered as a risk indicator for several metabolic diseases such as type 2 diabetes (T2D) and cardiovascular disease [73]. Therefore, future studies should investigate whether mannose levels can be used as biomarkers for other stress conditions, in addition to characterizing the physiological effects of mannose metabolism in the initiation, progress, and resolution of the stress response.

Finally, we identified several other metabolic changes (e.g., increased branched-chain amino acids (BCAA) and 5-aminovalerate) in the AS-exposed brain samples. BCAAs are key nitrogen donors in the glutamate/glutamine cycle. Within the central nervous system (CNS), glutamate, which is formed via BCAA transamination, is an excitatory neurotransmitter and substrate for the synthesis of the major inhibitory neurotransmitter GABA [74]. Current theories on the role of BCAAs in the brain are consistent with the involvement of glutamatergic and/or GABAergic systems in the etiology of neurological disorders. Fluctuations in BCAA levels significantly influence CNS function, particularly the balance between excitation and inhibition. BCAA metabolism contributes to the synthesis of new glutamate when this amino acid becomes depleted in the neurons in response to oxidative stress, and therefore, this process plays a crucial role in brain function homeostasis [75]. The levels of BCAA, leucine, isoleucine, and Val are decreased in patients suffering from psychiatric disorders such as major depressive disorder, immune-related major depression, and bipolar disorder [76,77,78]. BCAA levels are also elevated in the hippocampus after antidepressant treatment [79]. Furthermore, 5-aminovaleric acid, which is endogenously synthesized or derived from the metabolism of lysine by gut microbiota, is also known to act as a methylene homolog of gamma-aminobutyric acid (GABA) and functions as a weak GABA agonist [80]. Recently, 5-aminovaleric acid was identified in the plasma and brain tissues of Alzheimer’s disease patients [81]. It is currently unclear whether 5-aminovaleric acid could play a role in the stress response, presumably through synaptic modulation.

Our transcriptomic analyses identified a molecular profile that likely represents a short-term change in neural signaling and ROS metabolism. Overall, these gene expression changes demonstrate the weak relationship between the mRNA and metabolite levels. In turn, these findings suggest that there is increased regulation at an intermediate step between molecular events and metabolic reactions. As recently reported by von Ziegler et al. [33], the waves of increased and decreased transcriptional events upon acute WAS stimulation should be further investigated to fully understand the mechanisms through which the brain perceives acute stress and elicits homeostatic responses over time.

In conclusion, our study provides a detailed characterization of the molecular changes that occur in response to acute stress. Specifically, our study explored how a brief 1-h episode of water avoidance stress affected the transcription and metabolic profile of the brain in response to acute stress. Future studies will need to investigate region-specific changes using various omics technology platforms such as epigenomics and proteomics. Single-cell data with a higher temporal resolution will also be required to identify reliable stress biomarkers and gain insights into whether such molecular dysregulations could be associated with stress-related psychiatric disorders.

## Data Availability

Data available in a publicly accessible repository.

## Supplementary Materials

The following supporting information can be downloaded at: https://www.mdpi.com/article/10.3390/metabo13030453/s1, Table S1: List of 60 DEGs in water avoidance stress (WAS) versus the control; Table S2: List of significantly altered brain metabolites in acute WAS versus the control; Table S3: Summary of joint pathway integration analysis of transcriptomic and metabolomic data with Metaboanalyst 5.0.
