# Supplementary material for: Metabolic and Transcriptomic Signatures of the Acute Psychological Stress Response in the Mouse Brain
Source: Metabolites. 2023 Mar 20;13(3):453. doi: 10.3390/metabo13030453 (PMC10052811; doi:10.3390/metabo13030453)
Supplement: Supplementary file 1 [file metabolites-13-00453-s001.zip › metabolites-2257312-supplementary-done.pdf]

metabolites

Table S1. List of 60 DEGs in water avoidance stress (WAS) versus the control.

Gene Symbol	Description	Fold Change (Water Stress/Control)	*p* Value	Regulation
Satb2	special AT-rich sequence binding protein 2	1.621	0.014	Up
Pecr	peroxisomal trans-2-enoyl-CoA reductase	1.503	0.020	Up
Dnajb3	DnaJ heat shock protein family (Hsp40) member B3	0.619	0.015	Down
Serpinb8	serine (or cysteine) peptidase inhibitor, clade B, member 8	1.824	0.024	Up
C130074G19Rik	RIKEN cDNA C130074G19 gene	1.638	0.038	Up
Gucd1	guanylyl cyclase domain containing 1	1.540	0.001	Up
Smtn	smoothelin	0.640	0.043	Down
Emid1	EMI domain containing 1	1.789	0.007	Up
Doc2b	double C2, beta	0.658	0.007	Down
Grb7	growth factor receptor bound protein 7	0.627	0.043	Down
Hist1h1e	histone cluster 1, H1e	1.895	0.004	Up
A830009L08Rik	RIKEN cDNA A830009L08 gene	1.957	0.002	Up
Smim4	small integral membrane protein 4	0.666	0.001	Down
Atad2	ATPase family, AAA domain containing 2	1.585	0.001	Up
Igfbp6	insulin-like growth factor binding protein 6	1.682	0.019	Up
Serpind1	serine (or cysteine) peptidase inhibitor, clade D, member 1	0.638	0.007	Down
Zfp960	zinc finger protein 960	1.581	0.008	Up
Noxo1	NADPH oxidase organizer 1	0.597	0.047	Down
Epb4.1l4a	erythrocyte membrane protein band 4.1 like 4a	0.529	0.004	Down
Blnk	B cell linker	1.682	0.021	Up
Kcnh7	potassium voltage-gated channel, subfamily H (eag-related), member 7	1.510	0.047	Up
Adam33	a disintegrin and metallopeptidase domain 33	1.604	0.032	Up
Ovol2	ovo like zinc finger 2	1.604	0.043	Up
Pxmp4	peroxisomal membrane protein 4	0.647	0.000	Down
Nkain4	Na+ /K+ transporting ATPase interacting 4	0.621	0.002	Down
P2ry1	purinergic receptor P2Y, G-protein coupled 1	0.573	0.038	Down
Paqr6	progestin and adipoQ receptor family member VI	0.637	0.005	Down
Slc22a15	solute carrier family 22 (organic anion /cation transporter), member 15	0.631	0.016	Down
Fam212b	family with sequence similarity 212, member B	1.525	0.010	Up
Gstm6	glutathione S-transferase, mu 6	0.550	0.005	Down

Arsj	arylsulfatase J	0.563	0.030	Down
Tacr3	tachykinin receptor 3	0.532	0.050	Down
Adamtsl1	ADAMTS-like 1	0.651	0.030	Down
Ttc39aos1	Ttc39a opposite strand RNA 1	0.562	0.019	Down
Mycl	v-myc avian myelocytomatosis viral oncogene lung carcinoma derived	0.644	0.049	Down
Zbtb8a	zinc finger and BTB domain containing 8a	1.956	0.017	Up
Kit	kit oncogene	0.648	0.036	Down
Arhgap24	Rho GTPase activating protein 24	0.579	0.037	Down
Cabp1	calcium binding protein 1	1.522	0.015	Up
Nos1	nitric oxide synthase 1, neuronal	0.656	0.021	Down
Ncf1	neutrophil cytosolic factor 1	1.527	0.045	Up
Arhgap25	Rho GTPase activating protein 25	1.568	0.002	Up
Fancd2	Fanconi anemia, complementation group D2	1.598	0.021	Up
Zfand4	zinc finger, AN1-type domain 4	0.638	0.025	Down
C1ra	complement component 1, r subcomponent A	1.787	0.026	Up
Npas1	neuronal PAS domain protein 1	0.547	0.038	Down
Zfp658	zinc finger protein 658	0.662	0.002	Down
Dkkl1	dickkopf-like 1	1.533	0.030	Up
Cemip	cell migration inducing protein, hyaluronan binding	1.816	0.028	Up
B4galnt4	beta-1,4-N-acetyl-galactosaminyl transferase 4	0.658	0.023	Down
Gm10635	predicted gene 10635	2.413	0.022	Up
Zfp105	zinc finger protein 105	0.537	0.014	Down
Jade3	jade family PHD finger 3	0.585	0.049	Down
Igsf1	immunoglobulin superfamily, member 1	0.343	0.039	Down
Ccdc160	coiled-coil domain containing 160	0.560	0.010	Down
Heph	hephaestin	0.551	0.022	Down
Mum1l1	melanoma associated antigen (mutated) 1-like 1	0.615	0.001	Down
Usp51	ubiquitin specific protease 51	0.576	0.045	Down
2210013O21Rik	RIKEN cDNA 2210013O21 gene	0.666	0.029	Down
Adgrg2	adhesion G protein-coupled receptor G2	0.543	0.003	Down

Abbreviations: DEG, differentially expressed gene.

Table S2. List of significantly altered brain metabolites in acute WAS versus the control.

Super Pathway	Sub Pathway	Biochemical Name	Fold Change	*p* Value	Platform
Amino Acid	Lysine Metabolism	5-aminovalerate	2.16	0.0015	LC/MS pos
	Phenylalanine and Tyrosine Metabolism	homovanillate (HVA)	1.17	0.0319	LC/MS neg
	Leucine, Isoleucine and Valine Metabolism	leucine	1.17	0.0341	LC/MS pos
		isoleucine	1.18	0.0339	LC/MS pos
		ethylmalonate	1.29	0.0136	LC/MS polar
		valine	1.18	0.0286	LC/MS pos
	Glutathione Metabolism	4-hydroxy-nonenal-glutathione	1.21	0.0105	LC/MS neg
Carbohydrate	Pentose Phosphate Pathway	sedoheptulose-7-phosphate	1.19	0.0323	LC/MS neg
	Fructose, Mannose and Galactose Metabolism	mannose	2.94	0.0297	LC/MS polar
Lipid	Long Chain Fatty Acid	palmitate (16:0)	1.17	0.0165	LC/MS neg
		palmitoleate (16:1n7)	1.25	0.0409	LC/MS neg
		margarate (17:0)	1.19	0.0132	LC/MS neg
		stearate (18:0)	1.19	0.0369	LC/MS neg
		10-nonadecenoate (19:1n9)	1.32	0.0425	LC/MS neg
		eicosenoate (20:1n9 or 11)	1.27	0.0491	LC/MS neg
	Polyunsaturated Fatty Acid (n3 and n6)	eicosapentaenoate (EPA; 20:5n3)	1.31	0.0252	LC/MS neg
		docosapentaenoate (n3 DPA; 22:5n3)	1.38	0.0347	LC/MS neg
		linolenate [alpha or gamma; (18:3n3 or 6)]	1.49	0.0449	LC/MS neg
		dihomo-linolenate (20:3n3 or n6)	1.37	0.0021	LC/MS neg
		adrenate (22:4n6)	1.49	0.0246	LC/MS neg
		docosapentaenoate (n6 DPA; 22:5n6)	1.29	0.0238	LC/MS neg
		dihomo-linoleate (20:2n6)	1.31	0.0328	LC/MS neg
		mead acid (20:3n9)	1.74	0.0063	LC/MS neg
	Eicosanoid	prostaglandin F2alpha	1.29	0.0014	LC/MS neg
		15-HETE	1.29	0.011	LC/MS neg
	Endocannabinoid	palmitoyl ethanolamide	1.49	0.0196	LC/MS neg
		N-palmitoyltaurine	1.65	0.0333	LC/MS neg
	Phospholipid Metabolism	choline phosphate	1.06	0.0407	LC/MS pos
	Lysolipid	1-palmitoleoylglycerophosphocholine (16:1)*	0.68	0.0215	LC/MS pos
		1-oleoylglycerophosphoethanolamine	1.08	0.01	LC/MS polar
		1-arachidonoylglycerophosphoethanolamine*	1.23	0.0485	LC/MS neg
		1-palmitoylglycerophosphoinositol*	1.63	0.0013	LC/MS neg
		1-stearoylglycerophosphoinositol	1.55	0.0014	LC/MS neg
		1-arachidonoylglycerophosphoinositol*	1.49	0.0136	LC/MS neg

		1-stearoylglycerophosphoserine*	1.55	0.0008	LC/MS neg
		1-oleoylglycerophosphoserine	1.69	0.0005	LC/MS neg
		1-palmitoylglycerophosphoglycerol*	1.66	0.0154	LC/MS neg
		1-palmitoylglycerophosphoserine*	2.99	0.0016	LC/MS neg
		1-oleoylglycerophosphoglycerol*	1.75	0.0193	LC/MS neg
	Steroid	corticosterone	1.3	0.0332	LC/MS pos
Nucleotide	Purine Metabolism, Guanine containing	N2,N2-dimethylguanosine	1.58	0.0297	LC/MS pos
	Pyrimidine Metabolism, Cytidine containing	cytidine	1.17	0.0411	LC/MS pos
Xenobiotics	Food Component/Plant	methyl glucopyranoside (alpha + beta)	1.5	0.047	LC/MS pos

Table S3. Summary of joint pathway integration analysis of transcriptomic and metabolomic data with Metaboanalyst 5.0.

Pathway Name	Total	Expected	Hits	Raw *p*	−log10(*p* Value)	Holm Adjust	FDR	Impact
Biosynthesis of unsaturated fatty acids	47	0.39413	5	2.97E-05	4.5266	0.0024982	0.0024982	0.1087
Valine, leucine and isoleucine biosynthesis	12	0.10063	3	1.06E-04	3.9754	0.0087834	0.0044446	0.36364
Aminoacyl-tRNA biosynthesis	74	0.62055	3	0.022368	1.6504	1	0.59028	0.041096
Glycerophospholipid metabolism	86	0.72117	3	0.03312	1.4799	1	0.59028	0.17647
Valine, leucine and isoleucine degradation	88	0.73795	3	0.035136	1.4542	1	0.59028	0.08046
Arachidonic acid metabolism	79	0.66247	2	0.14032	0.85287	1	1	0.051282
alpha-Linolenic acid metabolism	22	0.18449	1	0.1698	0.77006	1	1	0.14286
Arginine biosynthesis	27	0.22642	1	0.20437	0.68958	1	1	0.15385
Glycosylphosphatidylinositol (GPI)-anchor biosynthesis	31	0.25996	1	0.23104	0.63631	1	1	0.13333
Pantothenate and CoA biosynthesis	34	0.28512	1	0.25048	0.60122	1	1	0.060606
Drug metabolism - cytochrome P450	39	0.32704	1	0.28185	0.54998	1	1	0.052632
Pentose phosphate pathway	47	0.39413	1	0.32946	0.48219	1	1	0.086957
Porphyrin and chlorophyll metabolism	53	0.44444	1	0.36319	0.43987	1	1	0.038462
Glutathione metabolism	56	0.4696	1	0.37944	0.42086	1	1	0.054545
Drug metabolism - other enzymes	69	0.57862	1	0.44542	0.35123	1	1	0.029412
Fatty acid elongation	75	0.62893	1	0.47357	0.32462	1	1	0.027027
Arginine and proline metabolism	78	0.65409	1	0.48713	0.31236	1	1	0.051948
Tyrosine metabolism	88	0.73795	1	0.52998	0.27574	1	1	0.022989
Pyrimidine metabolism	99	0.83019	1	0.57317	0.24171	1	1	0.030612
Fatty acid degradation	102	0.85535	1	0.58428	0.23338	1	1	0.019802
Metabolism of xenobiotics by cytochrome P450	117	0.98113	1	0.63583	0.19666	1	1	0.22414
Fatty acid biosynthesis	129	1.0818	1	0.67264	0.17222	1	1	0.015625
Steroid hormone biosynthesis	175	1.4675	1	0.78357	0.10592	1	1	0.028736
